# Plasma Metabolomic Profiles Associated with Three-Year Progression of Age-Related Macular Degeneration

**DOI:** 10.3390/metabo12010032

**Published:** 2022-01-01

**Authors:** Ines Lains, Kevin Mendez, Archana Nigalye, Raviv Katz, Vivian Paraskevi Douglas, Rachel S. Kelly, Ivana K. Kim, John B. Miller, Demetrios G. Vavvas, Liming Liang, Jessica Lasky-Su, Joan W. Miller, Deeba Husain

**Affiliations:** 1 Massachusetts Eye and Ear, Department of Ophthalmology, Harvard Medical School, Boston, MA 02114, USA; Ines_Lains@meei.harvard.edu (I.L.); Archana_Nigalye@meei.harvard.edu (A.N.); Raviv_katz@meei.harvard.edu (R.K.); Vivianparaskevi_douglas@meei.harvard.edu (V.P.D.); ivana_kim@meei.harvard.edu (I.K.K.); John_miller@meei.harvard.edu (J.B.M.); Demetrios_vavvas@meei.harvard.edu (D.G.V.); Joan_miller@meei.harvard.edu (J.W.M.); 2 Department of Biostatistics, Harvard T.H. Chan School of Public Health, Boston, MA 02114, USA; Kevin_mendez@meei.harvard.edu (K.M.); hprke@channing.harvard.edu (R.S.K.); Jessica.su@channing.harvard.edu (J.L.-S.); 3 Systems Genetics and Genomics Unit, Channing Division of Network Medicine, Brigham and Women’s Hospital and Harvard Medical School, Boston, MA 02114, USA; Lliang@hsph.harvard.edu

**Keywords:** age-related macular degeneration, dark adaptation, metabolomics, plasma

## Abstract

Plasma metabolomic profiles have been shown to be associated with age-related macular degeneration (AMD) and its severity stages. However, all studies performed to date have been cross-sectional and have not assessed progression of AMD. This prospective, longitudinal, pilot study analyzes, for the first time, the association between plasma metabolomic profiles and progression of AMD over a 3-year period. At baseline and 3 years later, subjects with AMD (*n* = 108 eyes) and controls (*n* = 45 eyes) were imaged with color fundus photos for AMD staging and tested for retinal function with dark adaptation (DA). Fasting plasma samples were also collected for metabolomic profiling. AMD progression was considered present if AMD stage at 3 years was more advanced than at baseline (*n* = 26 eyes, 17%). Results showed that, of the metabolites measured at baseline, eight were associated with 3-year AMD progression (*p* < 0.01) and 19 (*p* < 0.01) with changes in DA. Additionally, changes in the levels (i.e., between 3 years and baseline) of 6 and 17 metabolites demonstrated significant associations (*p* < 0.01) with AMD progression and DA, respectively. In conclusion, plasma metabolomic profiles are associated with clinical and functional progression of AMD at 3 years. These findings contribute to our understanding of mechanisms of AMD progression and the identification of potential therapeutics for this blinding disease.

## 1. Introduction

Age-related macular degeneration (AMD) remains the leading cause of blindness in people over the age of 50 years in developed countries [1]. Worldwide, it ranks third, and is expected to affect 288 million people by 2040 [1]. The hallmarks of the early phases of AMD include macular drusen and pigmentary changes. Some patients progress to the late forms of the disease, characterized by the presence of choroidal neovascularization (exudative form) or geographic atrophy (non-exudative form). Rates of progression to the advanced forms of AMD vary among individuals, with some patients remaining stable for years and others never developing late AMD. The reasons behind this variability remain poorly understood. Several clinical, genetic and environmental factors (such as fundus features, risk single nucleotide polymorphisms and smoking, respectively) have been linked to risk of AMD progression [2]. However, their interlinks and functional consequences remain insufficiently characterized [3]. This has important consequences for clinical care because (i) there are currently limited strategies for halting AMD progression and for treating non-exudative forms of AMD; [3] moreover, (ii) the current standard of care for assessment of risk of AMD progression remains solely based on fundus appearance by examination or imaging, and it is limited in its prediction ability. Researchers have tried to combine AMD status at baseline with genetic variants and demographic and environmental factors to build models of risk prediction [4], but their application remains limited [5]. 

Our group hypothesized that plasma metabolomics, the qualitative and quantitative analysis of metabolites (<1–1.5 KDa), could contribute in addressing these challenges by providing insights into the pathophysiology of AMD [6]. Metabolites are the downstream product of cumulative effects of the genome and its interaction with environmental exposures; therefore, the metabolome is thought to be closely related to disease phenotype, especially in multifactorial diseases [7]. Indeed, we [8,9,10] and others [11] have described that patients with AMD have a distinct plasma metabolomic profile than compared to controls, and this profile changes with disease severity. To our knowledge, however, no studies have evaluated how plasma metabolomic profiles relate to AMD progression. This pilot study aimed to analyze the association between plasma metabolomic profiles and progression of AMD over a three-year period. In addition to the conventional outcome of phenotypic progression based on color fundus photographs, we also assessed associations with rod-mediated dark adaptation, which is a promising functional AMD biomarker [12,13].

## 2. Results

We included data on 153 eyes of 81 patients. Table 1 presents clinical and demographic characteristics of the included study population. As shown, 17% of the eyes (*n* = 26) demonstrated progression of AMD stage at the 3-year visit. This included nine eyes that at baseline were classified as controls and progressed to either early (*n* = 5) or intermediate AMD (*n* = 4) at 3 years; eight eyes with early AMD at baseline that progressed to intermediate AMD; and nine eyes with intermediate AMD at baseline that developed late AMD during the 3-year follow-up period. Among the nine eyes that developed late AMD, eight progressed to geographic atrophy and one developed choroidal neovascularization.

### 2.1. Metabolomic Profiles and AMD Progression Based on Color Fundus Photographs

Eight of the baseline metabolites showed a significant association with AMD progression at 3 years (*p* < 0.01)-Table 2. Among them, the most significant metabolites (ENT80) were ribitol (ß = 7.76, *p* = 0.0002) and pregnenediol disulfate (C21H34O8S2) (ß = −1.82 × 10^15^, *p* = 0.0014). Pathway analysis revealed a significant enrichment of the pentose and glucuronate interconversions pathway (*p* = 0.004).

When looking at changes in metabolite levels over the 3-year period, six metabolites showed a significant association with AMD progression, and one of them (ascorbic acid 2-sulfate, ß = 4.58, *p* = 0.0016) was significant based on ENT80—Table 3.

### 2.2. Metabolomic Profile and Changes in Dark Adaptation

For 38 eyes of 24 patients included in the study, we had available data on dark adaptation both at baseline and at the 3-year follow-up visit. Table 4 presents their characterization. 

Of the baseline metabolites, none presented significant associations (all *p* > 0.01) with changes in RIT at 3 years. When looking at changes of metabolites at 3 years, a sphingomyelin (hydroxypalmitoyl sphingomyelin (d18:1/16:0(OH)), ß = 11.7, *p* = 0.0091) presented a significant association.

**Figure 1:**
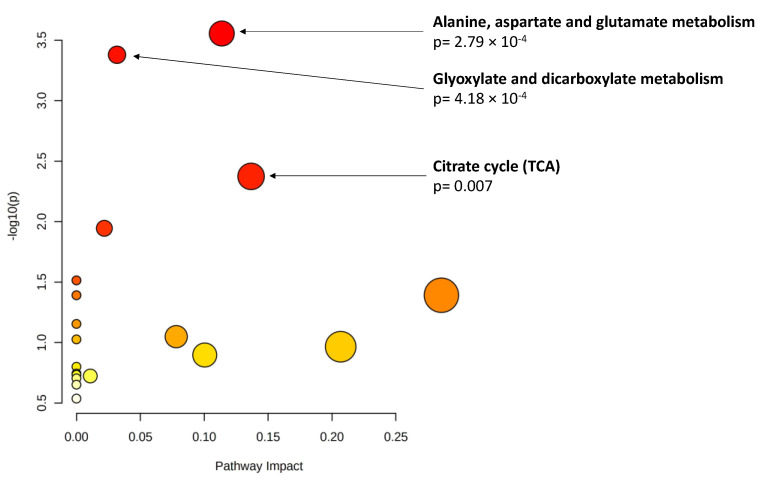
Pathway analysis of baseline metabolites associated with changes in area under the dark adaptation curve (AUDAC) at 3 years (*p* < 0.01). *X* axis: pathway impact, calculated as the sum of the importance measures of the matched metabolites normalized by the sum of the importance measures of all metabolites in each pathway. *Y* axis: −log(p): logarithm of the *p*-value, based on the hypergeometric pathway enrichment test. The color and size of each circle are based on its *p*-value and pathway impact value, respectively. Namely, colors vary from white to red from less significant to most significant *p*-values.

As explained, an important percentage of the eyes included in this study had reached the RIT ceiling test value at the time of inclusion (*n* = 9, 23.7% of the eyes with data on DA); thus, we used AUDAC as an additional outcome. Among the baseline metabolites, 19 showed a significant association (*p* < 0.01, Table 5) with changes in AUDAC at 3 years. Glutamine (ß = −0.18, *p* = 0.0012) and taurocholenate sulfate (ß = 0.19 *p* = 0.0012) reached statistical significance based on the ENT80 cutoff. Pathway analysis of the 19 metabolites pointed to an enrichment of three metabolomic pathways (*p* < 0.01, Figure 1).

**Figure 2:**
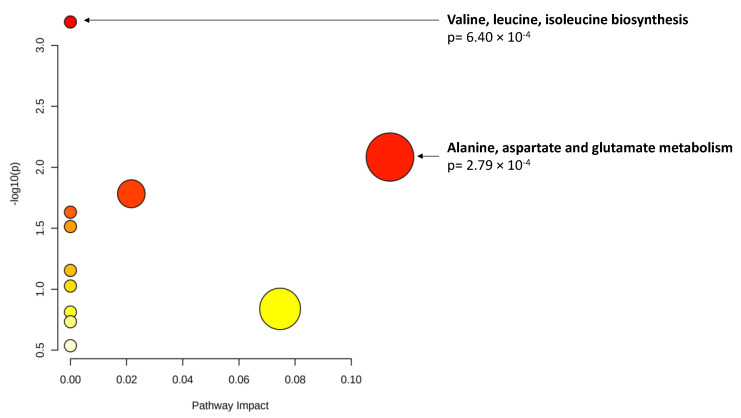
Pathway analysis of metabolite changes (*p* < 0.01) associated with area under the dark adaptation curve (AUDAC) changes at 3 years. *X* axis: pathway impact, calculated as the sum of the importance measures of the matched metabolites normalized by the sum of the importance measures of all metabolites in each pathway. *Y* axis: −log(p): logarithm of the *p*-value, based on the hypergeometric pathway enrichment test. The color and size of each circle are based on its *p*-value and pathway impact value, respectively. Namely, colors vary from white to red from less significant to most significant *p*-values.

Finally, when considering changes in metabolite levels at 3 years, 17 metabolites demonstrated significant associations (*p* < 0.01) with changes in AUDAC at 3 years. Among them, glutamine showed statistical significance when accounting for multiple comparisons. These results are presented in Table 6. Pathway analysis (Figure 2) revealed a significant enrichment of two pathways (*p* < 0.01): valine, leucine and isoleucine biosynthesis; and alanine, aspartate and glutamate metabolism.

## 3. Discussion

We present a prospective longitudinal study on the association of plasma metabolomic profiles and progression of AMD at a structural and functional level. Despite its small sample size and, therefore, pilot nature, to our knowledge, this is the first longitudinal evaluation of metabolomic profiles in AMD. Our results revealed that both baseline levels and 3-year changes of metabolites in pentose and glucoronate interconversions pathway and pregnenolone steroids were associated with AMD progression based on color fundus photographs. Additionally, when using dark adaptation as a functional outcome, we observed that baseline and changes in glutamine and in alanine and aspartate and glutamate pathways were associated with 3-year changes in the area under the dark adaptation curve (AUDAC). 

The association of metabolites in the pentose and glucoronate interconversions pathway, such as ribitol, with progression of AMD is an interesting finding, and the finding points to the role of oxidative stress in the pathogenesis of this disease. Studies in human retinal pigment epithelium (RPE) cell lines have shown that, when the pentose phosphate pathway is disturbed, the antioxidant capacity of the RPE cells is impaired [15]. The human RPE is highly specialized and has essential functions to both the photoreceptors and the choriocapillaris [16]. However, it is particularly prone to oxidative stress because of the high levels of light exposure, oxygen supply by the choroidal vasculature and metabolic activity of the photoreceptors [17]. Under physiologic conditions, there are efficient repair mechanisms in RPE to compensate for the highly oxidized environment. When these are abnormal, however, RPE and photoreceptors cell death ensues. Indeed, oxidative stress and reduced antioxidant capacity are considered hallmarks of the pathogenesis of AMD progression [17,18].

Our findings on associations between levels of androgenic and pregnanediol steroids and AMD progression at 3 years also point to the role of oxidative stress in this disease. Sex hormones are not only produced in the gonads but also in the central nervous system (CNS), [19] including in the retina [20]. The retinal pathway starts by the synthesis of cholesterol, which is then converted in pregnenolone, which results in the formation of progestin and androgen metabolites and then estrogen [21]. Sex steroid hormone receptors (SSHRs) have also been identified in the retina, [20] where estrogens are believed to have an antioxidant and anti-inflammatory role, [22,23] protecting against retinal degeneration [21]. Testosterone, progesterone and their metabolites also appear capable of modulating neurotransmission and may participate in controlling the sex steroid milieu of the retina [21]. Additionally, sex hormones may have an influence in retinal function by modulating retinal and choroidal blood flow [24]. While estrogens exert a vasodilatory effect, progesterone and androgenic steroids have opposite effects [21]. Despite the current understanding of the basic mechanisms of action of hormonal steroids in the retina, there is still important controversy clinically, as clinical and epidemiological studies have added several dimensions of complexity. For example, while some investigators have reported that exposure to estrogens (including exogenous estrogens) can be a protective factor against developing subretinal drusenoid deposits and neovascular AMD, [25] other studies did not confirm these associations [21]. Additionally, a meta-analysis published by Chakravarthy et al. [26] has shown that gender does not have a consistent association with AMD.

In this study, we also assessed associations between plasma metabolomic profiles and changes in rod-mediated dark adaptation, which is a promising functional outcome in AMD. Consistent data have shown that DA impairment correlates with different stages of disease [13,27] and that more advanced baseline AMD stages are associated with more pronounced changes in DA over time [28,29]. Thus, we were interested in assessing, in this cohort, how plasma metabolomic profiles associate with changes in DA at 3 years. RIT is the conventional outcome for DA testing. However, in our study, similarly to most cohorts of patients with AMD, an important percentage of eyes had reached the RIT ceiling test value (20 min) at baseline, thus precluding longitudinal evaluation. By focusing on area under the dark adaptation curve, a recent alternative outcome proposed by our group, we were able to include all eyes with DA data. Our results consistently showed that glutamine was associated with changes in AUDAC at 3 years, with pathway analysis pointing to an enrichment of metabolites in alanine, aspartate and glutamate pathways. Interestingly, our group has recently published a cross-sectional study [30] that also showed that amino acids related to glutamate are among those with the most significant associations with AUDAC (and also RIT). 

These findings are not surprising in light of the current understanding of plasma metabolomic changes in AMD and also on the role of glutamate in rod apoptosis. Consistent studies have shown that changes in metabolites in the glutamate pathway are observed in AMD. Namely, both our studies using nuclear magnetic resonance spectroscopy (NMR) [8] and mass spectrometry (MS) [10] have pointed to a dysregulation of metabolites in alanine, aspartate and glutamate pathways in patients with AMD and across different stages of disease. Using targeted metabolomics, Kersten et al. [31] also reported that the levels of glutamine were different in the aqueous humor of patients with early and intermediate AMD compared to controls. Additionally, previous literature has proposed explanations for why dysregulations in glutamine and associated pathway metabolites may play a role in DA impairments. Glutamine is considered the most abundant and versatile amino acid in the human body [32]. It can be used for the synthesis of nucleotides (purines, pyrimidines and amino sugars), nicotinamide adenine dinucleotide phosphate (NAPDH) and antioxidants (such as glutathione) involved in the maintenance of cellular integrity and function [33]. Glutamate is also an important excitatory neurotransmitter for the visual pathway, including the retina. However, through excitotoxicity, it can result in neural cell damage or death, [34] which is thought to be mediated at least in part by oxidative stress [35]. Indeed, Charles-Messance et al. [36] showed in a mouse model that glutamate alone is sufficient for inducing rod photoreceptor cell death and that this is due to the deregulation of glutamate homeostasis and glutamate excitotoxicity.

The main limitation of this study is its small sample size, especially in analyses considering dark adaptation outcomes. However, to our knowledge, this is the first time that a longitudinal evaluation of metabolomic profiles in AMD was performed, providing opportunities for further research. With this relatively small sample size, we were unable to evaluate associations with progression to the two subtypes of late AMD–choroidal neovascularization and geographic atrophy—which likely have different pathogenic mechanisms. Due to our sample size, we report *p*-values < 0.01, which increases the risk of false positive results considering the number of tests performed in this study. Given this limitation, we also provided results based on the ENT80 significance thresholds. Additionally, our follow-up for now is limited to 3 years, while AMD is a disease characterized by progression over 5 years to decades. Studies with longer longitudinal evaluations are needed in order to better establish the primary mechanisms of AMD progression. In this manuscript, we based our assessment of AMD progression on color fundus photographs, which remain the gold-standard method for this disease. However, color photographs have important limitations and do not reflect the wide spectrum of AMD phenotypes that is currently well recognized and particularly well appreciated with optical coherence tomography (OCT). In particular, specific OCT features, such as the presence of subretinal drusenoid deposits, have been linked to AMD progression and dark adaptation, and we did not assess their presence in this study. Finally, even though all samples used in this study were collected after confirmed overnight fasting and we focused on endogenous metabolites, diet and nutritional parameters are known to affect metabolomic profiles and we did not include them in our analyses. 

## 4. Materials and Methods

This was a prospective longitudinal observational study that took place at the Massachusetts Eye and Ear (Mass Eye and Ear), Harvard Medical School, Boston, Massachusetts. The clinical protocol was conducted in accordance with HIPAA (Health Insurance Portability and Accountability Act) requirements and the tenets of the Declaration of Helsinki and was approved by the Institutional Review Board of the Massachusetts Eye and Ear/Massachusetts General Brigham (Protocol number 14–111H and 2019P002725). All subjects enrolled in the study provided written informed consent. 

### 4.1. Study Population

As previously reported, [9,10] from January 2015 to July 2016, we recruited subjects diagnosed with AMD, as well as control subjects with no evidence of AMD and aged ≥ 50 years from the Mass Eye and Ear Retina Service and Comprehensive Ophthalmology and Optometry Services. Exclusion criteria included the following: diagnosis of any other vitreoretinal disease; active uveitis or ocular infection; significant media opacities that precluded the observation of the ocular fundus; refractive error equal or greater than 6 diopters of spherical equivalent; past history of retinal surgery; history of any ocular surgery or intra-ocular procedure (such as intra-ocular injections) within the 90 days prior to enrolment; and diagnosis of diabetes mellitus. As described below, for this study, patients with late AMD at baseline were also excluded.

Three years later (±3 months), the same individuals were invited to participate in a follow-up visit. The same exclusion criteria applied, with the exception of a history of recent intra-ocular procedures because we were also interested in patients that could have developed late neovascular AMD. 

### 4.2. Study Protocol

The study procedures were the same for both the baseline visit and the 3-year follow-up and have been described in detail [9,10]. Briefly, all included participants received a complete bilateral ophthalmologic examination and were imaged with 7-field, non-stereoscopic color fundus photographs (CFP) using either a Topcon TRC-50DX (Topcon Corporation, Tokyo, Japan) or a Zeiss FF-450Plus (Carl Zeiss Meditec, Dublin, CA, USA) camera. Additionally, complete medical history was obtained, which included self-reported data on smoking habits, and patients were invited to perform dark adaptation testing according to the protocol described below. 

For all participants, fasting venous blood samples were collected into a sodium-heparin tube, which was centrifuged within 30 min (1500 rpm, 10 min, 20 °C) to obtain plasma for metabolomic analysis. Plasma aliquots of 1.5 mL were then transferred into sterile cryovials and stored at −80 °C. For the baseline visit, as patients were recruited during their regular ophthalmic appointments, an additional visit had to be frequently scheduled for blood collection in order to ensure overnight fasting. This was scheduled within a maximum of 1 month after study inclusion. For the 3-year follow-up visit, the patients were contacted in advance; thus, fasting blood collection usually took place on the same day of the remaining study procedures. 

### 4.3. Dark Adaptation Testing

Dark adaptation testing (DA) was optional because it required additional time from the participants. In order to avoid prior light exposure, DA was performed on a separate day than retinal imaging, within a maximum time limit of 1 month after enrollment in the study (both for baseline and for 3-year follow-up). Our protocol has been described previously in detail [13]. Briefly, we evaluated DA using the AdaptDx^®^ dark adaptometer (MacuLogix, Harrisburg, PA, USA) extended protocol (20 min) [27]. Sensitivity was estimated using a modified staircase threshold estimate procedure, with an initial stimulus intensity of 5 scot cd/m^2^. The test ended when the patient’s sensitivity recovered by 3.0 log units (corresponding to the level of 5 × 10^−3^ scot cd/m^2^) or the test duration reached 20 min, whichever came first. The machine then estimates the slope of the second component of rod-mediated dark adaptation and extrapolates the time required for sensitivity to recover by 3.0 log units, which is designated as rod-intercept time (RIT). For analysis, RIT data were exported and eyes with fixation errors ≥ 30% were excluded. 

Additionally, data on successive threshold measurements were exported to calculate the area under the dark adaptation curve (AUDAC) [37]. This is a recent alternative outcome that our group has developed and proposed because an important percentage of patients with AMD reached the ceiling value of DA testing (in the case of our protocol 20 min); thus, it was not possible to evaluate their changes longitudinally [37]. We used the standard trapezoidal method, [38] considering area under the curve from the start of the DA test (time = 0, sensitivity threshold = 0) to the time when the sensitivity threshold of 3.0 log units was achieved. Linear interpolation was used when the sensitivity threshold was not exactly at 3.0 log units [39]. When the curve did not reach the 3.0 log unit threshold, the 20 min testing time limit was used. In order to allow for easier interpretation and comparison between subjects, the area computed was then normalized by the maximum possible area between 0 and 20 min limits along the *x* axis and 0 to 3.0 log unit limits along the *y* axis. Therefore, AUDAC was expressed as a percentage of the overall area, with larger values indicating delays in DA, elevated sensitivity threshold or both. 

### 4.4. AMD Staging and Definition of Progression

Field 2 CFPs were standardized by using software developed by our group [40] and then graded on IMAGEnet 2000 software according to the Age-related Eye Disease Study (AREDS) 2 classification system [41]. The following groups were established: [41,42] control group (AREDS level 1)—presence of drusen maximum size < circle C0 and total area < C1; early AMD (AREDS level 2)—drusen maximum size ≥ C0 but <C1 or presence of AMD characteristic pigment abnormalities in the inner or central subfields; intermediate AMD (AREDS level 3)—presence of drusen maximum size ≥ C1 or drusen maximum size ≥ C0 if the total area occupied is >I2 for soft indistinct drusen and >O2 for soft distinct drusen; and late AMD (AREDS level 4)—presence of GA according to the criteria described above or evidence of neovascular AMD. The baseline images were graded as part of our initial study [13]. Eyes that at baseline had late AMD were not considered for analyses (*n* = 43 eyes) because we were interested in those that could progress over the 3-year period. For CFP obtained at the 3-year follow-up visit, the same protocol was followed. In cases of disagreement, the senior author established the final categorization. 

### 4.5. Metabolomic Profiling and Data Processing

Plasma samples were shipped to Metabolon, Inc^®^. in dry ice. The samples arrived frozen in less than 24 h and were immediately stored at −80 °C until processing. Non-targeted MS analysis was performed using Ultrahigh Performance Liquid Chromatography-Tandem MS (UPLC-MS/MS) according to previously published protocols [9]. Samples were analyzed in three batches: baseline samples from a pilot study, [9] the remaining baseline samples [10] and three-year visit samples. Batches were then merged using bridge sample normalization [43] based on Metabolon’s long-term reference quality control samples. Metabolites that were not present in 50% of the quality control samples in all three batches could not be merged; thus, they were removed.

Metabolite data were subsequently run through our standard quality control and data processing pipeline [6]. Missing values were imputed with half the minimum observed value in all samples for each metabolite [44]. Metabolites features were then log-transformed to reduce heteroscedasticity (obtained approximately normal distributions) [45] and then pareto scaled to reduce the influence of metabolites with very high levels while keeping data structure partially intact. These steps are important to allow for a standardized comparison of metabolite levels [45]. In order to ensure that only the most informative metabolites were included in the analyses, metabolites with interquartile range levels of zero were excluded [6]. Additionally, we excluded metabolites that are exogenous to humans (e.g., medications, food additives and buffering agents) from subsequent analyses, as we were interested in investigating endogenous metabolites that could be driving systemic biology. Finally, we only considered subjects with missing or undetected metabolite percentages less than 30%. Based on this criterion, however, no participants were excluded, as the highest percentage of missing values by subject was 10.4%. Thus, the final analyses included 643 endogenous metabolites, among which the majority had either zero (*n* = 418) or a low proportion (<10%) of missing values (*n* = 176). By using principal component analysis (PCA), we identified one sample as a possible outlier, which was then excluded.

### 4.6. Statistical Analysis

Descriptive statistics were used to summarize clinical and demographic characteristics of the included study population, including mean and standard deviation for continuous variables and percentages for dichotomous/categorical variables. 

In order to analyze the association between plasma metabolite levels and AMD progression, we used multilevel mixed-effect logistic models to account for the inclusion of both eyes of the same patient. By definition, these models are appropriate for research designs where data for participants are organized at more than one level (i.e., nested data). In this study, the units of analysis were considered the eyes (at a lower level), which are nested within patients’ contextual/aggregate units (at a higher level) [46]. In these models, AMD progression was defined as any change at three-years in AMD status based on color fundus photographs (i.e., control to any AMD stage, early AMD to intermediate or late AMD or intermediate to late AMD). By using AMD progression as the outcome, we performed two analyses: (i) baseline metabolite levels as the exposure and (ii) change in metabolite levels between three years and baseline as the exposure.

As we were also interested in the association between plasma metabolite levels and changes in dark adaptation over three years, we used multilevel mixed-effect linear regression by using the previous approach with RIT change and AUDAC change at three years as the outcome. For the models with RIT changes as the outcome, only eyes that at baseline were able to reach RIT within the 20 min of testing were considered, as it was not possible to demonstrate gradual worsening relative to eyes that at baseline had already reached the ceiling test value. All reported models accounted for age, gender, smoking, BMI and AMD stage as covariates.

For all analyses, we report *p*-values at two thresholds: *p*-values < 0.01 to denote a trend towards significance, and *p*-values < 0.0016 to denote statistically significant findings after accounting for multiple testing. The statistically significant threshold (*p*-value < 0.0016) was calculated based on the effective number of independent tests accounting for 80% variance (ENT80) [47,48]. Additionally, for all reported *p*-values we provide beta coefficients. Beta coefficients represent change in the outcome variable for AMD progression or changes in RIT/AUDAC for one unit of change in the predictor variable (while holding other predictors in the model constant, such as the confounding variables we accounted for) [49]. For example, this means that, for the models with AMD progression as outcome, for one unit change in metabolite levels, the odds of AMD progressing increase by the value of the beta coefficient. For the models with RIT/AUDAC as outcomes (continuous variables), beta coefficients refer to change in RIT/AUDAC per change in levels of metabolites. All analyses were conducted in R, version 4.0.3.

When possible, pathway analyses on the significant metabolites identified was performed to further interpret the biological relevance of our findings. This was achieved by using Metaboanalyst 4.0 [50], which combines overrepresentation analysis with topology analysis to identify pathways that are dysregulated based on (i) the number of metabolites from our significant metabolites that fall within KEGG-defined metabolic pathways and (ii) the positional importance of our metabolites within these pathways. These analyses generate a pathway impact score and associated *p*-value.

## 5. Conclusions

In conclusion, this pilot study suggests that baseline plasma metabolites and changes in plasma metabolites are associated with AMD progression at 3 years. In particular, while several metabolites and metabolomic pathways were identified in this study, and all of them have a converging biological link to oxidative stress. This suggests that oxidative stress may play a particularly important role in the progression of AMD both at a structural and functional level. Further studies with larger sample sizes and longer follow-up periods are required to clarify the results of this pilot study and, hopefully, to help identify potential druggable targets for this blinding disease. 

## Figures and Tables

**Table 1:** Demographics of the included study population.

	AMD Progression	No AMD Progression	Total
Eyes, *n* (%)	26 (17)	127 (83)	153 (100)
Baseline AMD stage, *n* (%) Control Early AMD Intermediate AMD	9 (34.6) 8 (30.8) 9 (34.6)	36 (28.3) 15 (11.8) 76 (59.9)	45 (29.4) 23 (15.0) 85 (55.6)
Age, mean ± SD	71.35 ± 5.75	69.30 ± 6.88	69.61 ± 6.73
Female, *n* (%)	19 (73.1)	95 (74.8)	114 (74.5)
BMI, mean ± SD	24.66 ± 3.21	27.23 ± 4.67	26.80 ± 4.55
Smoking, *n* (%) Ex-smoker Non-smoker Smoker	17 (65.4) 7 (26.9) 2 (7.7)	62 (48.9) 61 (48.0) 4 (3.1)	79 (51.6) 68 (44.4) 6 (3.9)
Race, *n* (%) White Black Hispanic	24 (92.3) 2 (7.7) 0 (0)	122 (96.1) 2 (1.6) 3 (2.4)	146 (95.4) 4 (2.6) 3 (2.0)

Legend: AMD–age-related macular degeneration, *n*–number, SD–standard deviation and BMI–body mass index.

**Table 2:** Baseline metabolites associated with progression of AMD based on color fundus photographs (*p* < 0.01).

Metabolite	Super Pathway	Sub Pathway	ß Coefficient	*p* Value
N6,N6,N6-trimethyllysine	Amino Acid	Lysine Metabolism	4.929	0.0083
Phenylalanine	Amino Acid	Phenylalanine Metabolism	−3.02 × 10^15^	0.0052
Methylsuccinate	Amino Acid	Leucine, Isoleucine and Valine Metabolism	4.938	0.0088
N-methylhydroxyproline *	Amino Acid	Urea cycle; Arginine and Proline Metabolism	−2.108	0.0068
Ribitol	Carbohydrate	Pentose Metabolism	−7.755	0.0002 ^a^
N-palmitoyl-sphingosine (d18:1/16:0)	Lipid	Ceramides	−9.442	0.0052
Pregnenediol disulfate (C21H34O8S2) **	Lipid	Pregnenolone Steroids	−1.82 × 10^15^	0.0014 ^a^
1-linoleoyl-2-linolenoyl-GPC (18:2/18:3) *	Lipid	Phosphatidylcholine (PC)	−3.476	0.0046

Legend: Data of 153 eyes were considered for this analysis. ^a^ Statistically significant based on ENT80, *p* < 0.0016. * Metabolite with level 2 of identification [14]. ** Metabolite with level 3 identification [14].

**Table 3:** Changes in metabolites at 3 years and associations with progression of AMD based on color fundus photographs (*p* < 0.01).

Metabolite	Super Pathway	Sub Pathway	ß Coefficient	*p*-Value
Methylsuccinate	Amino Acid	Leucine, Isoleucine and Valine Metabolism	−2.665	0.0088
Ribonate	Carbohydrate	Pentose Metabolism	−3.652	0.0026
Ascorbic acid 2-sulfate	Cofactors and Vitamins	Ascorbate and Aldarate Metabolism	4.584	0.0016 ^a^
5alpha-androstan-3alpha,17beta-diol monosulfate (1)	Lipid	Androgenic Steroids	8.381	0.0025
5alpha-androstan-3beta,17beta-diol monosulfate (2)	Lipid	Androgenic Steroids	9.206	0.0099
Androstenediol (3beta,17beta) monosulfate (1)	Lipid	Androgenic Steroids	13.480	0.0068
Pregnenetriol disulfate **	Lipid	Pregnenolone Steroids	1.82 × 10^15^	0.0026

Legend: Data of 153 eyes were considered for this analysis. ^a^ Statistically significant based on ENT80, *p* < 0.0016. ** Metabolite with level 3 identification [14].

**Table 4:** Demographics of the included eyes with data available on dark adaptation.

	AMD Progression	No AMD Progression	Total
Eyes, *n* (%)	9 (24)	29 (76)	38 (100)
Baseline AMD stage Control Early AMD Intermediate AMD	6 (66.7) 1 (11.1) 2 (22.2)	7 (24.1) 1 (3.5) 21 (72.4)	13 (34.2) 2 (5.3) 23 (60.5)
Age	67.6 ± 3.4	67.8 ±7.0	67.7 ± 6.3
Female, *n*(%)	7 (77.8)	17 (58.6)	24 (63.2)
BMI	24.1 ± 3.0	26.6 ± 3.5	26.0 ± 3.5
Smoking Ex-smoker Non-smoker Smoker	5 (55.6) 3 (33.3) 1 (11.1)	16 (55.2) 13 (44.8) 0 (0)	21 (55.3) 16 (42.1) 1 (2.6)
Race White Black Hispanic	7 (77.8) 2 (22.2) 0 (0)	27 (93.1) 0 (0) 2 (6.9)	34 (89.5) 2 (5.3) 2 (5.3)

Legend: AMD–age-related macular degeneration; *n*—number; SD—standard deviation; BMI—body mass index.

**Table 5:** Baseline metabolites associated with changes in area under the dark adaptation curve (AUDAC) at 3 years.

Metabolite	Super Pathway	Sub Pathway	ß Coefficient	*p*-Value
glutamine	Amino Acid	Glutamate Metabolism	−0.1789	0.0012 ^a^
3-methyl-2-oxobutyrate	Amino Acid	Leucine, Isoleucine and Valine Metabolism	−0.2154	0.0052
isovalerylcarnitine (C5)	Amino Acid	Leucine, Isoleucine and Valine Metabolism	−0.1303	0.0093
cysteine sulfinic acid	Amino Acid	Methionine, Cysteine, SAM and Taurine Metabolism	0.1499	0.0046
P-cresol glucuronide *	Amino Acid	Tyrosine Metabolism	−0.0799	0.0068
3-amino-2-piperidone	Amino Acid	Urea cycle; Arginine and Proline Metabolism	0.1404	0.0025
pyruvate	Carbohydrate	Glycolysis, Gluconeogenesis, and Pyruvate Metabolism	−0.2190	0.0066
N-acetylneuraminate	Carbohydrate	Aminosugar Metabolism	0.1221	0.0053
N-acetylglucosamine/N-acetylgalactosamine	Carbohydrate	Aminosugar Metabolism	0.0922	0.0080
Gulonate *	Cofactors and Vitamins	Ascorbate and Aldarate Metabolism	0.1844	0.0030
citrate	Energy	TCA Cycle	−0.2426	0.0033
5alpha-pregnan-3beta,20alpha-diol disulfate	Lipid	Progestin Steroids	−0.2872	0.0044
taurocholenate sulfate *	Lipid	Secondary Bile Acid Metabolism	0.1879	0.0012 ^a^
5alpha-pregnan-3beta,20beta-diol monosulfate (1)	Lipid	Progestin Steroids	−0.2761	0.0020
palmitoylcholine	Lipid	Fatty Acid Metabolism (Acyl Choline)	−0.0827	0.0031
Linoleoylcholine *	Lipid	Fatty Acid Metabolism (Acyl Choline)	−0.0854	0.0029
gamma-glutamylglutamine	Peptide	Gamma-glutamyl Amino Acid	−0.1125	0.0048
gamma-glutamylhistidine	Peptide	Gamma-glutamyl Amino Acid	−0.1711	0.0082
prolylglycine	Peptide	Dipeptide	−0.1284	0.0092

Legend: Data of 38 eyes were considered for this analysis. ^a^ Statistically significant based on ENT80, *p* < 0.0016. * Metabolite with level 2 identification [14].

**Table 6:** Changes in metabolites at 3 years and associations with changes in area under the dark adaptation curve (*p* < 0.01).

Metabolite	Super Pathway	Sub Pathway	ß Coefficient	*p*-Value
glutamine	Amino Acid	Glutamate Metabolism	0.1748	0.0013 ^a^
asparagine	Amino Acid	Alanine and Aspartate Metabolism	0.1443	0.0079
4-methyl-2-oxopentanoate	Amino Acid	Leucine, Isoleucine and Valine Metabolism	0.2286	0.0089
3-methyl-2-oxobutyrate	Amino Acid	Leucine, Isoleucine and Valine Metabolism	0.1861	0.0032
hydantoin-5-propionate	Amino Acid	Histidine Metabolism	0.1868	0.0019
3-amino-2-piperidone	Amino Acid	Urea cycle; Arginine and Proline Metabolism	−0.1144	0.0088
N-acetylglucosamine/N-acetylgalactosamine	Carbohydrate	Aminosugar Metabolism	−0.0953	0.0043
biliverdin	Cofactors and Vitamins	Hemoglobin and Porphyrin Metabolism	−0.1158	0.0070
alpha-tocopherol	Cofactors and Vitamins	Tocopherol Metabolism	0.2110	0.0094
sphingomyelin (d18:2/14:0, d18:1/14:1) *	Lipid	Sphingomyelins	0.1138	0.0076
palmitoylcholine	Lipid	Fatty Acid Metabolism (Acyl Choline)	0.0703	0.0047
lactosyl-N-nervonoyl-sphingosine (d18:1/24:1) *	Lipid	Lactosylceramides (LCER)	0.1897	0.0057
glycosyl ceramide (d18:2/24:1, d18:1/24:2) *	Lipid	Hexosylceramides (HCER)	0.2083	0.0060
Linoleoylcholine *	Lipid	Fatty Acid Metabolism (Acyl Choline)	0.0709	0.0020
glycosyl ceramide (d18:1/20:0, d16:1/22:0) *	Lipid	Hexosylceramides (HCER)	0.1065	0.0088
gamma-glutamylglutamine	Peptide	Gamma-glutamyl Amino Acid	0.1106	0.0094
gamma-glutamylglycine	Peptide	Gamma-glutamyl Amino Acid	0.1482	0.0052

Legend: Data of 38 eyes were considered for this analysis. ^a^ Statistically significant based on ENT80, *p* < 0.0016. * Metabolite with level 2 identification [14].

## Data Availability

All data can be made available upon request. The data are not publicly available due to ongoing study and data collection including longer time point and new baseline data.
